# Exome Sequencing in Monogenic Forms of Rickets

**DOI:** 10.1007/s12098-022-04393-9

**Published:** 2023-01-24

**Authors:** Prince Jacob, Gandham SriLakshmi Bhavani, Prajna Udupa, Zheng Wang, Sankar V. Hariharan, Kishan Delampady, Ashwin Dalal, Nutan Kamath, Shiro Ikegawa, Rathika D. Shenoy, Koushik Handattu, Hitesh Shah, Katta M. Girisha

**Affiliations:** 1 Department of Medical Genetics, Kasturba Medical College, Manipal, Manipal Academy of Higher Education, Manipal, Karnataka India; 2 Laboratory for Bone and Joint Diseases, RIKEN Center for Integrative Medical Sciences, Tokyo, Japan; 3 Department of Pediatrics, Genetics Clinic, SAT Hospital, Government Medical College, Thiruvananthapuram, Kerala India; 4 Department of Endocrinology, AJ Hospital & Research Center, Mangalore, Karnataka India; 5 Diagnostics Division, Center for DNA Fingerprinting & Diagnostics, Hyderabad, Telangana India; 6 Department of Pediatrics, Kasturba Medical College, Mangalore, Manipal Academy of Higher Education, Manipal, Karnataka India; 7 Department of Pediatrics, K S Hegde Medical Academy, Nitte University, Mangalore, Karnataka India; 8 Department of Pediatrics, Kasturba Medical College, Manipal, Manipal Academy of Higher Education, Manipal, Karnataka India; 9 Department of Pediatric Orthopedics, Kasturba Medical College, Manipal, Manipal Academy of Higher Education, Manipal, Karnataka India

**Keywords:** Rickets, Vitamin-D-dependent rickets, Hypophosphatemic rickets, Exome sequencing

## Abstract

**Objective:**

To understand the phenotypic and genotypic spectrum of genetic forms of rickets in 10 families.

**Methods:**

Detailed clinical, radiographic, and biochemical evaluation of 10 families with phenotypes suggestive of a genetic cause of rickets was performed. Molecular testing using exome sequencing aided in the diagnosis of six different forms of known genetic causes.

**Results:**

Eleven disease-causing variants including five previously reported variants (*CYP27B1*:c.1319_1325dup, p.(Phe443Profs*24), *VDR*:c.1171C>T, p.(Arg391Cys), *PHEX*: c.1586_1586+1del, *PHEX:* c.1482+5G>C, *PHEX:* c.58C>T, p.(Arg20*)) and six novel variants (*CYP27B1*:c.974C>T, p.(Thr325Met), *CYP27B1:* c.1376G>A, p.(Arg459His), *CYP2R1*: c.595C>T, p.(Arg199*), *CYP2R1:*c.1330G>C, p.(Gly444Arg),*SLC34A3*:c.1336-11_1336-1del, *SLC2A2*: c.589G>C, p.(Val197Leu)) in the genes known to cause monogenic rickets were identified.

**Conclusion:**

The authors hereby report a case series of individuals from India with a molecular diagnosis of rickets and provide the literature review which would help in enhancing the clinical and molecular profile for rapid and differential diagnosis of rickets.

**Supplementary Information:**

The online version contains supplementary material available at 10.1007/s12098-022-04393-9.

## Introduction

Rickets is a disorder of growing bone caused by a deficiency of calcium/phosphorous/vitamin D or defects in their metabolism. Defective mineralization and widening of the cartilaginous growth plates are the characteristic features of rickets [1]. Since the first description of the term “rickets” in the medical literature [2], nutritional deficiency has been the most prevalent cause of rickets worldwide. Among nutritional deficiencies, vitamin D deficiency is reported as a common etiology [3]. In India, the prevalence rate ranges from 22%–92% in infants and 14%–24% in older children [4]. However, there are other non-nutritional causes of rickets including genetic causes, rickets due to drug ingestion, secondary rickets due to liver and kidney diseases, and malabsorption syndromes [5].

Hereditary forms of rickets are very rare and account for only about 13% [6]. Based on the underlying pathophysiology, they have been broadly classified into two categories, vitamin-D-dependent rickets (VDDR) and hypophosphatemic rickets (HR). VDDR is further subclassified into four different forms including VDDR1A, VDDR1B, VDDR2A, and VDDR2B, whereas hypophosphatemic rickets consists of 15 distinct disorders which are grouped into FGF-23-dependent and FGF-23-independent hypophosphatemic rickets [7]. More recently VDDR type 3 (VDDR3) caused by disease-causing variants in *CYP3A4* has been reported [8]. Hitherto, 19 genes are known to cause monogenic forms of rickets [7, 9]. The overlap of the clinical spectrum of hereditary rickets with nutritional deficiency poses a challenge to diagnosis [9]. Therefore, molecular diagnosis is crucial for rickets persisting after adequate treatment of underlying nutritional deficiency and thereby initiating appropriate treatment. In this study, the authors detail the clinical and mutational spectrum of VDDR and hypophosphatemic rickets in 10 Indian families along with their laboratory findings. This condition is not well studied in this population. Understanding the landscape of phenotype and genetic etiology would help in differential diagnosis at an early stage.

## Materials and Methods

Ten unrelated individuals from different families with suspected hereditary rickets were recruited for the study. A detailed history of illness, clinical photographs, and radiographs were obtained after receiving written informed consent from the families. An appropriate biochemical investigation was performed on all the subjects and values along with reference ranges were recorded. The Institutional Ethics Committee (IEC) of Kasturba Medical College and Hospital, Manipal granted ethical clearance (IEC:921/2018) for this study.

Exome sequencing (ES) was carried out in probands of all the families (Supplementary Fig. S1) using multiple sequencing platforms and capture kits as previously described [10]. Variant analysis was performed using a well-defined in-house strategy and pathogenicity assessment was based on parameters including type of variant, genomic location, effect on protein, patterns of inheritance, clinical correlation, allele frequency in population databases (gnomAD, and ExAC), in-house database (2155 exomes), and prediction scores of multiple in silico tools (including MutationTaster, REVEL, M-CAP, SIFT, Splice AI). Guidelines and criteria issued by the American College of Medical Genetics and Genomics and the Association of Molecular Pathologists (ACMG-AMP) were followed for classifying the disease-causing variants [11].

## Results

Ten unrelated Indian individuals (P1 to P10), their parents, and unaffected siblings from 10 families were recruited for the study. Consanguinity was observed in 7 families. Their ages at clinical diagnosis ranged from 2–23 y. Detailed clinical and molecular information of affected individuals and families along with their laboratory investigation results are provided in Supplementary Material S1, Supplementary Table S1 and S2, and Table 1. All the variants identified in this study were submitted to ClinVar database (accession numbers: SUB10956971,SUB10957212, SUB10957214, SUB10957222, SUB10957254, SUB10957263,SUB10957267, SCV002053826.1, SCV002054009, SCV002053849.1). The summary of clinical, biochemical, and molecular findings of all the individuals based on the final diagnosis are enumerated below:

**Table 1:** Summary of affected individuals with molecular diagnosis of rickets

Family ID	Family 1	Family 2	Family 3	Family 4	Family 5	Family 6	Family 7	Family 8	Family 9	Family 10
Subject ID	P1	P2	P3	P4	P5	P6	P7	P8	P9	P10
**Subject characteristics**										
Age	4 y	4 y	2 y	3 y 6 mo	4 y	22 y	10 y	23 y	13 y	8 y
Gender	Male	Female	Female	Female	Male	Female	Female	Female	Male	Female
Consanguinity	+	+	−	+	+	+	−	−	+	+
Ethnicity	Asian Indian	Asian Indian	Asian Indian	Asian Indian	Asian Indian	Asian Indian	Asian Indian	Asian Indian	Asian Indian	Asian Indian
Height in cm (SDS)	71 (-7 SDS)	87 (-2.5)	68 (−3)	NA	76 (−6)	145.7 (−2.6)	124.5 (−2.45)	127 (−5.2)	128 (−3.5)	80 (−9.6)
Weight in kg (SDS)	8.5 (-7 SDS)	11 (-2.2)	6.38 (−3)	NA	−	44 (−2.14)	24 (−2)	39 (−2.9)	30 (−4)	11.3 (−3.675)
Occipitofrontal circumference in cm (SDS)	47 (-3 SDS)	48.5 (-1.1)	44 (−2.5)	NA	47 (−2.4)	53.5 (−1)	52 (−1.33)	52.5 (−1.6)	50.5 (−3)	45.5 (−3)
**Clinical features**										
Wrist widening	+	+	+	+	+	−	+	+	+	+
Pectus carinatum	-	+	+	−	−	−	−	−	−	−
Pigeon chest	-	-	+	−	+	−	−	−	−	−
Harisson sulcus	-	-	+	−	+	−	−	−	−	−
Rachitic rosary	-	-	+	−	+	−	−	−	−	−
Pot belly	+	+	+	−	+	−	−	−	−	−
Double malleoli	+	-	+	−	+	−	−	−	−	−
Genu varum	+	-	+	+	+	+	+	+	−	−
Genu valgum	-	+	+	−	−	−	−	−	+	+
Pes planus	+	+	−	+	NA	+	+	+	+	NA
**Radiological features**										
Osteopenia	-	+	+	+	+	+	+	+	+	+
Delayed carpal bone ossification	+	-	+	−	+	−	−	−	−	−
Small epiphyses	+	+	+	+	+	−	−	−	−	−
Frayed and/or irregular metaphyses	-	+	+	+	+	+	+	+	+	+
Bowing of lower limbs	+	+	−	+	+	+	+	+	+	−
Bowing of upper limbs	-	-	−	NA	+	−	−	−	−	+
Additional features	Square shape face, broad nasal bridge, maxillary prominence, coarse facies, hypertelorism and oligodontia	Dolichocephaly	Bronchopneumonia, bilateral lower limb hypotonia	None	Carious teeth, sparse eyebrows, joint laxity, and lumbar kyphosis	Telecanthus, bushy eyebrows, synorphis, low set ears, esotropia, central corneal opacity with iridotomy in eyes, unilateral vision loss, bilateral hearing loss, and everted left foot	None	None	None	Mild blue sclearae, sparse eyebrows, multiple sites of fracture
**Molecular testing details**										
Gene(s)	*CYP27B1*	*CYP27B1*	*CYP27B1*	*CYP2R1*	*VDR*	*PHEX*	*PHEX*	*PHEX*	*SLC34A3*	*SLC2A2*
Transcript ID	NM_000785.4	NM_000785.4	NM_000785.4	NM_024514.4	NM_000376.3	NM_000444.6	NM_000444.6	NM_000444.6	NC_000009.12	NM_000340.2
Zygosity	Homozygous	Homozygous	Compound heterozygous	Compound heterozygous	Homozygous	Heterozygous	Heterozygous	Heterozygous	Homozygous	Homozygous
Disease-causing variants	c.974C>T	c.1319_1325dup	c.1376G>A; c.1319_1325dup	c.595C> T;c.1330G>C	c.1171C>T	c.1586_1586+1del	c.1482+5G > C	c.58C>T	c.1336-11_1336-1del	c.589G>C
Protein change	p.(Thr325Met)	p.(Phe443Profs*24)	p.(Arg459His); p.(Phe443Profs*24)	p.(Arg199*);p.(Gly444Arg)	p.(Arg391Cys)	−	−	p.(Arg20*)	−	p.(Val197Leu)
Location	Exon 6	Exon 8	Exon 8	Exon 3; Exon 4	Exon 11	Intron 14	Intron 13	Exon 1	Intron 12	Exon 5
Variant status	Novel	Known	1 novel variant	2 novel variants	Known	Known	Known	Known	Novel	Novel
OMIM disease	Vitamin-D dependent rickets type 1A	Vitamin-D dependent rickets type 1A	Vitamin-D dependent rickets type 1A	Vitamin-D dependent rickets type 1B	Vitamin-D dependent rickets type 2A	X-linked dominant hypophosphatemic rickets	X-linked dominant hypophosphatemic rickets	X-linked dominant hypophosphatemic rickets	Hypophosphatemic rickets with hypercalciuria	Fanconi–Bickel syndrome
ACMG classification (assertation criteria)	Variant of uncertain significance (PM2, PP3, PP4)	Pathogenic (PVS1, PM2, PP3, PP4, PP5)	Likely pathogenic (PM2, PM5, PP3, PP5) Pathogenic (PVS1, PM2, PP3, PP4, PP5)	Variant of uncertain significance (PM2, PP3, PP4)	Pathogenic (PS3, PM1, PM5, PM2, PP3, PP4)	Pathogenic (PVS1, PM2, PP5)	Likely pathogenic (PM2, PM4, PP4, PP5)	Pathogenic (PVS1, PM2, PP5)	Pathogenic (PVS1, PM2, PP4)	Likely pathogenic (PM2, PM5, PP3, PP4)

(+): Present, (−): Absent

*NA* Not available, *SDS* Standard deviation score

**Fig. 1:**
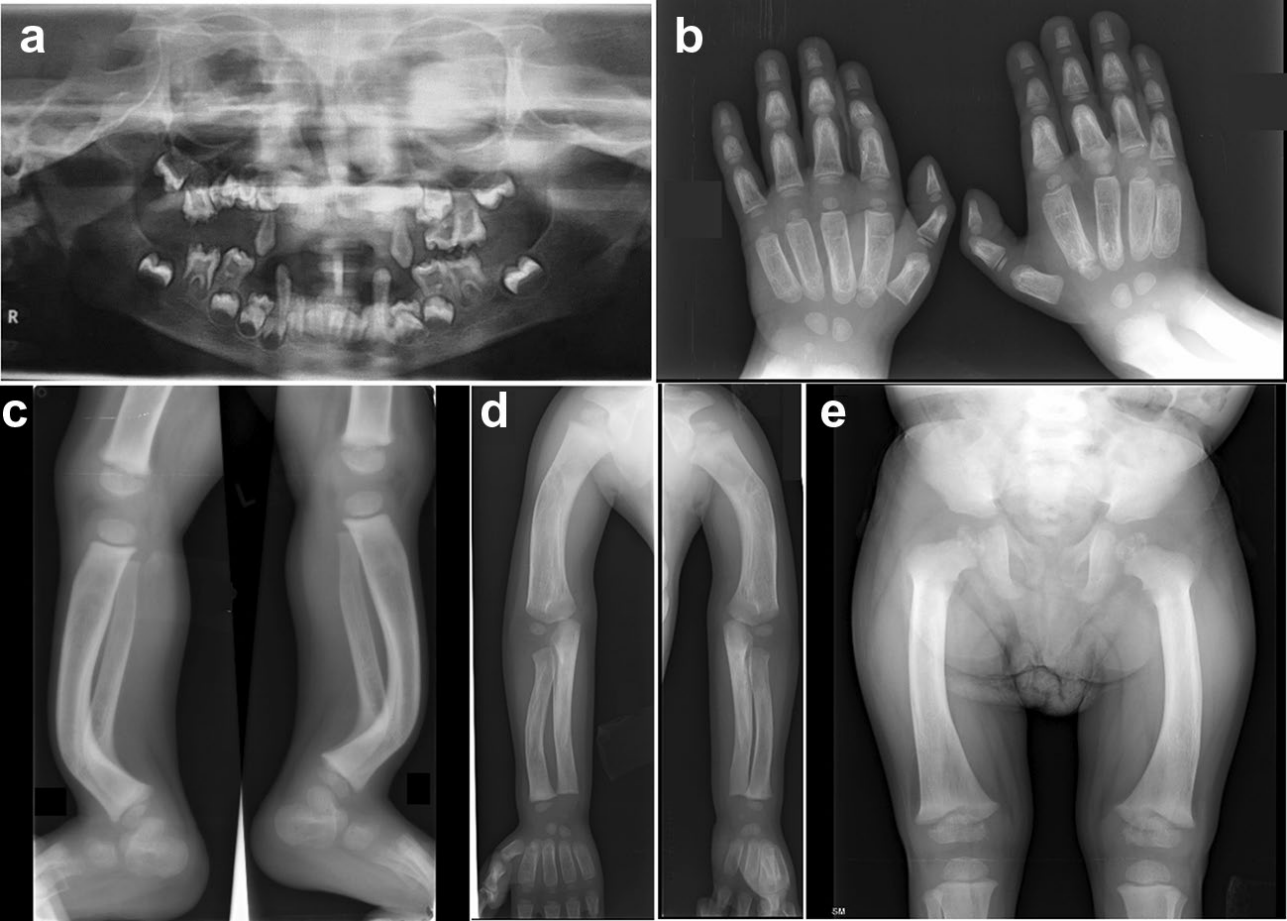
Radiographic profile of an individual with vitamin-D-dependent rickets type I. P1 (age: 4 y) shows delayed tooth eruption at 8 y 9 mo **(a)**, delayed carpal ossification **(b)**, metaphyseal dysplasia at the ends of long bones **(c–e)**: small epiphyses at the knee **(c)**, bending of long bones **(c, d)**, and small capital femoral epiphysis **(e)**. The radiographic appearance was affected by his treatment

*Vitamin-D-dependent rickets type 1A (VDDR1A):* Three affected individuals (P1, P2, and P3) from unrelated families had clinical and radiological features suggestive of VDDR1A. P1 had a severe spectrum of disease (Fig. 1) with early age of onset as compared to P2 and P3. The complete biochemical investigations were available for P1 and P3, which were consistent with the findings of VDDR1A. P3 had normal levels of vitamin D and calcium owing to the treatment with vitamin-D supplements. ES helped in the identification of variants in the homozygous state, c.974C>T in *CYP27B1* in P1, c.1319_1325dup in *CYP27B1* in P2, and variants in the compound heterozygous state, c.1376G>A, and c.1319_1325dup in *CYP27B1* in P3. Thus, a molecular diagnosis of VDDR1A was ascertained.

*Vitamin-D-dependent rickets type 1B (VDDR1B):* One affected individual (P4) was presented with bowing of legs and had radiological features suggestive of rickets. Her biochemical investigation revealed low serum calcium and phosphate levels with increased ALP values. Molecular analysis aided in the identification of two novel variants in the compound heterozygous state c.595C>T and c.1330G>C in *CYP2R1*, thus, a diagnosis of VDDR1B was ascertained.

**Fig. 2:**
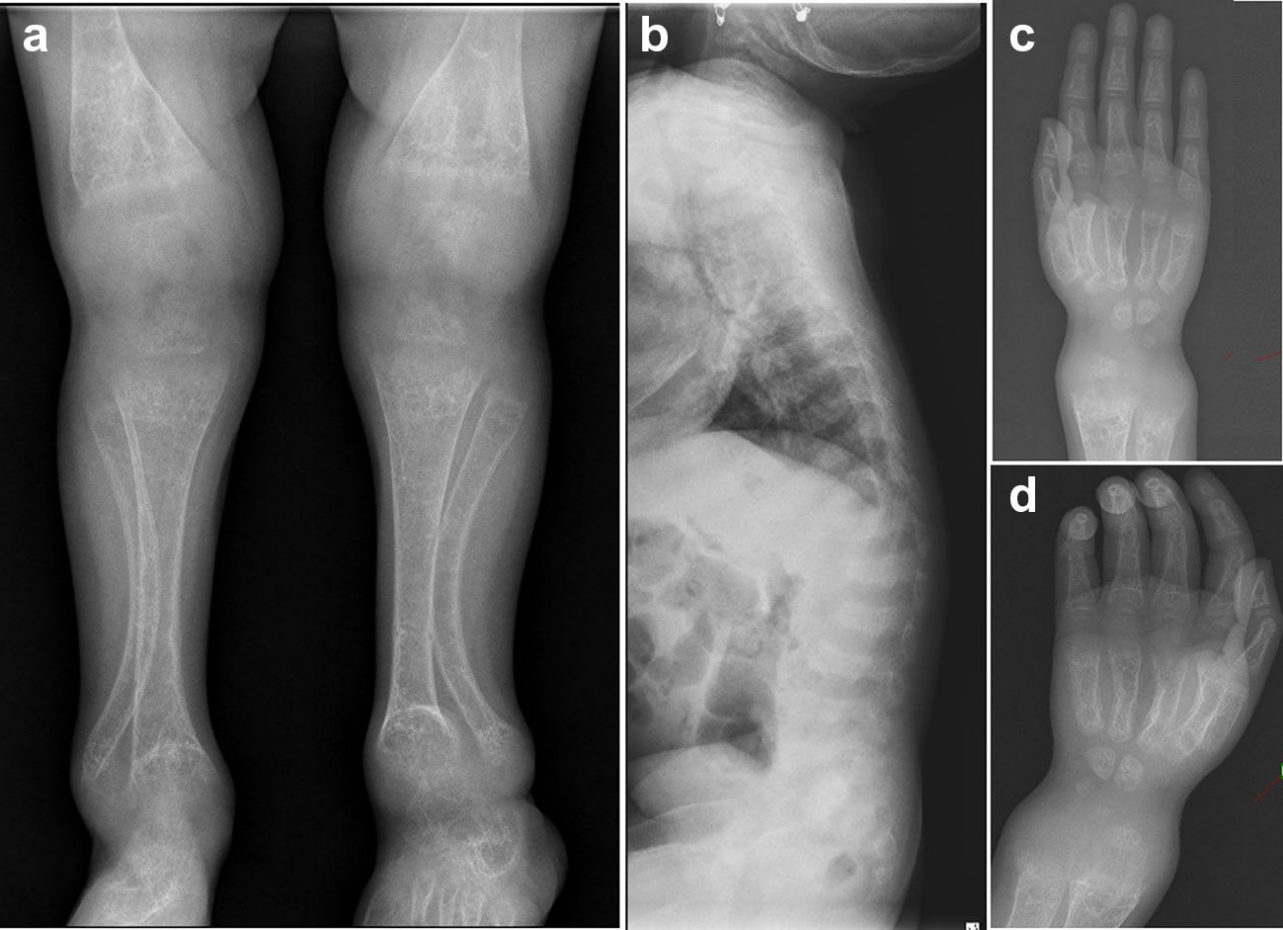
Radiographs of an individual with vitamin-D-dependent rickets type IIA. P5 (age 4 y) shows severe osteopenia, small epiphyses at the knee, irregular metaphyses, bending of the fibula **(a)**, dorsal–lumbar kyphosis **(b)**, and cupping of radius and ulna **(c **and** d)**

*Vitamin-D-dependent rickets type 2A (VDDR2A):* An affected individual (P5) was presented with a leg deformity and wrist widening (Fig. 2). He had a severe phenotype of rickets along with alopecia which was noticed since birth. His biochemical profile showed hypocalcemia, hypophosphatemia, and elevated ALP levels. Analysis of exome sequencing data led to the identification of a known missense variant, c.1171C>T in the homozygous state in VDR thereby asserting the diagnosis of VDDR2A.

**Fig. 3:**
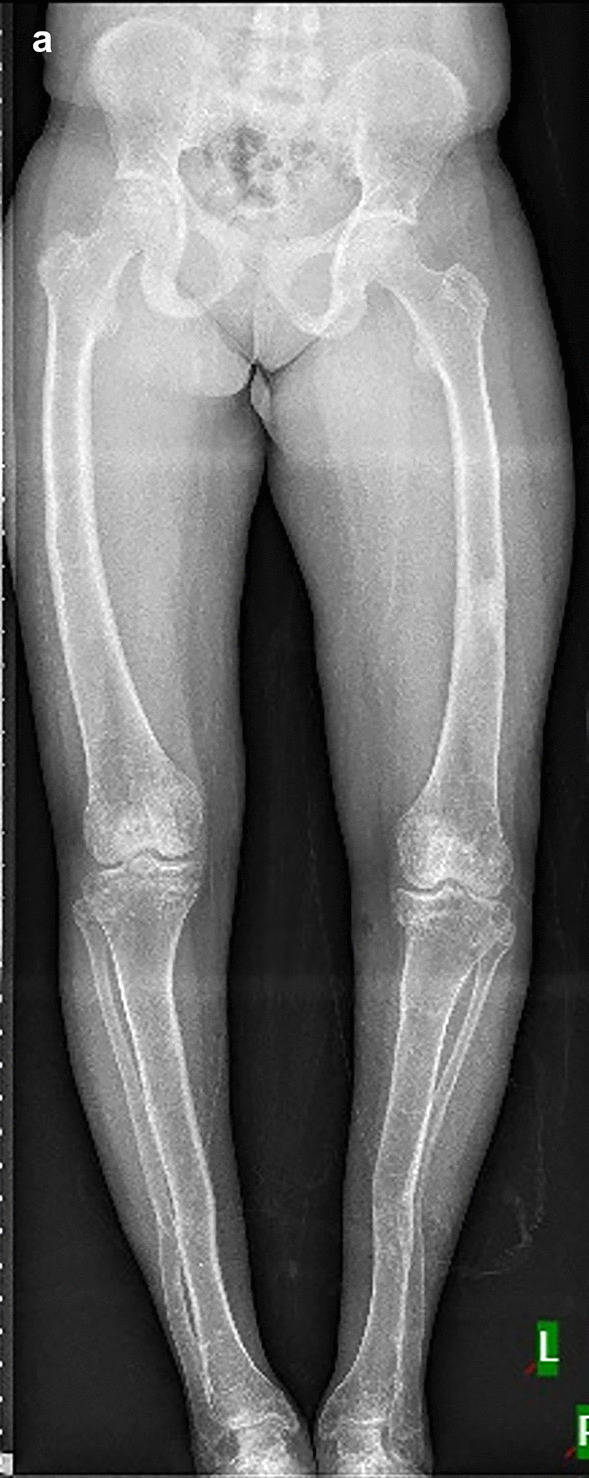
Lower limb radiograph of P6 with X-linked hypophosphatemic rickets (age 22 y). Bowing of long bones, and osteopenia with frayed metaphyses are seen **(a)**

*X-linked dominant hypophosphatemic rickets (XLDHR):* Three individuals (P6, P7, and P8) with clinical features suggestive of hypophosphatemic rickets were presented with genu varum and abnormal gait. One of the individuals (P6) had a severe phenotype (Supplementary Material S1 and Fig. 3) with early onset of disease. The biochemical findings were available for P6; however, low levels of serum phosphate and increased values of ALP were observed in P7 and P8. On molecular analysis, one splice-site variant, c.1586_1586+1del in *PHEX* was observed in the heterozygous state in P6. The c.1482+5G>C in *PHEX* was observed in P7, whereas c.58C>T was observed in heterozygous in *PHEX* in P8. Thus, a diagnosis of X-linked dominant hypophosphatemic rickets in all three individuals was made.

*Hypophosphatemic rickets with hypercalciuria (HHRH):* One individual (P9) was presented with short stature and difficulty in walking. His radiographs and biochemical findings (low phosphate levels with elevated ALP) were suggestive of a monogenic form of hypophosphatemic rickets. Analysis of ES data revealed a novel variant c.1336-11_1336-1del in the homozygous state in *SLC34A3*, thereby the molecular diagnosis was identified.

**Fig. 4:**
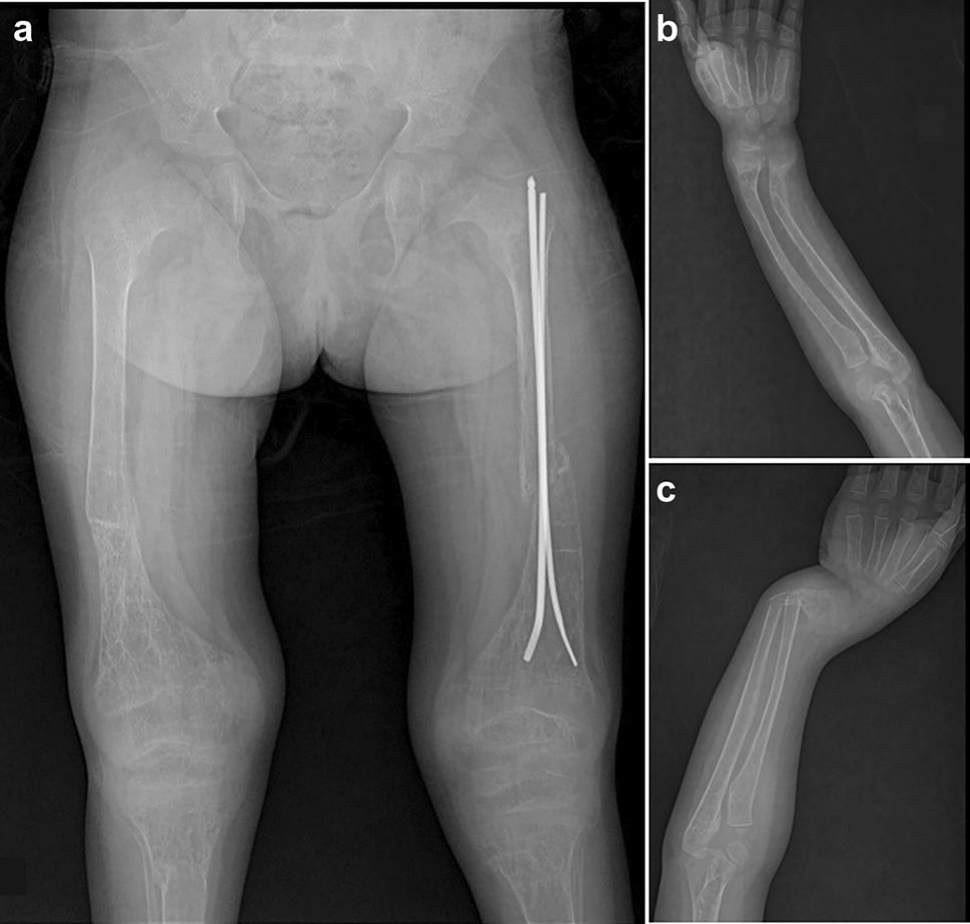
Radiographic profile of P10 (age 8 y) with Fanconi–Bickel syndrome. Multiple sites of fracture and osteopenia are seen in her upper and lower limbs **(a**–**c)**

*Fanconi–Bickel syndrome (FBS):* One affected girl (P10) of 8 y of age was referred with complaints of multiple fractures. Generalized osteopenia and bowing of upper limbs were observed in her radiographs (Fig. 4). Low levels of glucose, phosphate, and elevated serum ALP were observed in her laboratory profile. A provisional diagnosis of osteogenesis imperfecta was made. However, exome sequencing helped in the identification of a novel missense variant, c.589G>C in *SLC2A2*, which led to the diagnosis of FBS.

## Discussion

The interplay of multiple genetic factors maintains the metabolic homeostasis of vitamin D, calcium, and phosphate in the body, and the perturbation of this essential balance leads to different genetic forms of rickets. Disease-causing variants in *CYP27B1* cause an autosomal recessive disease, vitamin-D-dependent rickets type 1A, VDDR1A (MIM # 264700) [12]. The three most reported variants in *CYP27B1 *are c.1319_1325dup, c.262delG, and c.195+2T>G [13]. The most common variant, c.1319_1325dup is also found in two individuals in the present cohort (P2 and P3). Genotype–phenotype correlation for VDDR1A is not well established. However, by statistical analysis of reported disease-causing variants and clinical features, a recent study has highlighted a few findings [13]. According to this study, c.195+2T>G leads to the most severe clinical presentation whereas in contrast patients with c.262delG variant present with the least severe phenotypic manifestation owing to age and height at evaluation. However, affected individuals with variant, c.1319_1325dup (as found in P2 and P3) had different phenotypic manifestations according to a study. One of the individuals had seizures along with the clinical presentation and another unrelated individual had all the other typical manifestations of VDDR1A without seizures [14]. All the affected individuals (P1, P2, and P3) in the present study had biochemical profiles similar to the findings of VDDR1A [7] (Supplementary Table S1). Three affected individuals are described in the present study with variants in *CYP27B1*, one of the individuals (P1) showed the complete and severe spectrum of disease with enamel hypoplasia and hypocalcemic seizures with early age of onset (4 mo of age). Interestingly, the novel variant identified in P1 was nonsynonymous as compared to truncating variant found in the other two individuals (P2 and P3). The difference in disease severity could be possibly due to phenotypic variability [14].

Vitamin-D-dependent rickets type 1B, VDDR1B (MIM # 600081) is an autosomal recessive disease caused by biallelic variants in *CYP2R1* [15]. Only 6 variants in 31 patients have been described in literature hitherto [16]. Through the present study, the authors add two more novel variants in the literature, c.595C>T and c.1330G>C, which are identified in compound heterozygous state in *CYP2R1* in P4. Recently, Bakhamis et al. suggested a semi-dominant inheritance pattern of this disease affecting individuals equally with both biallelic and single-heterozygous variants [16]. The only clinical feature different in both groups of individuals was hypocalcemic manifestations. The typical biochemical findings of VDDR1B (except for PTH and 25-OH vitamin D levels) corroborate with the parameters of the affected subject (P4) in the present study (Supplementary Table S1) [7].

The third entity of the VDDR group of disorders, vitamin-D-dependent rickets type 2A, VDDR2A (MIM # 277440), is an autosomal recessive disease caused by biallelic variants in the vitamin D receptor gene, *VDR* [17]. VDR constitutes two functional domains, namely the N-terminal dual zinc finger DNA binding domain (DBD), and the C-terminal ligand-binding domain (LBD) [17]. Genotype–phenotype correlation in VDDR2A suggests that all the inactivating biallelic variants found in the DBD result in severe clinical presentation (with alopecia) due to complete loss of function whereas a milder phenotype is observed in individuals with variants in the LBD due to partial loss of function of VDR [18, 19]. However, there are exceptions to this rule [20]. The affected individual (P5) in the present cohort also had a severe spectrum (with alopecia) of disease presentation; however, the variant was located in LBD. Functional analysis performed for this variant revealed that Arg391Cys leads to poor VDRE binding in vivo and reduced transactivation thus leading to a severe phenotype [20]. The biochemical findings (except for PTH and 25-OH vitamin D levels) observed in the affected boy (P5) in the present study corroborate with the laboratory profile of VDDR2A (Supplementary Table S1) [7].

X-linked dominant hypophosphatemic rickets, XLDHR (MIM # 307800) belongs to the FGFR3-dependent rickets category, and it is caused by pathogenic variants in *PHEX* (phosphate-regulating endopeptidase homolog X-linked). A recent study has stated that individuals harboring pathogenic variants in the N-terminal region have an earlier onset of disease as compared to subjects with variants in C-terminus [21]. Nevertheless, the affected individuals (P6, P7, and P8) in the present cohort had similar ages of onset of disease (average: 2 y) irrespective of the location of variants (2 variants in C terminus and 1 variant in N terminus). The authors report three known truncating variants, c.1482+5G>C, c.1586_1586+1del and c.58C>T in *PHEX* reported earlier to cause XLDHR [22–24]. Both the canonical and noncanonical splice variants cause the skipping of exons 13 and 14, respectively. The phenotype observed in P6 harboring the canonical splice variant was comparatively more severe (Supplementary Material S1) than in the other two individuals (P7 and P8). The biochemical findings of affected individuals (P7 and P8) with variants in *PHEX* are mentioned in Supplementary Table S1.

Hypophosphatemic rickets with hypercalciuria, HHRH (MIM # 241530) is an autosomal recessive disease caused due to biallelic variants in *SLC34A3* (solute carrier family 34, member 3) [25]. A review of all the previously reported patients shows that affected individuals with rickets have markedly low levels of serum phosphate as compared to subjects with only renal phenotype. Additionally, most individuals with homozygous variants have rickets as compared to subjects with compound heterozygous variants whose phenotype is variable [26]. The affected individual (P9) in the present cohort harbored a novel variant, c.1336-11_1336-1del (in *SLC34A3*) in the homozygous state with all the characteristic features of HHRH including clinical and biochemical profiles (Table 1 and Supplementary Table S1) [7]. Subjects with a single-heterozygous variant (carriers) have also been reported earlier with mild symptoms [26].

Hypophosphatemia is a common feature of many other diseases which are not considered to be a classical form of HR. Fanconi–Bickel syndrome, FBS (MIM # 227810) an autosomal recessive disease is one such entity that mimics hypophosphatemic rickets as well as osteogenesis imperfecta (OI) [27]. FBS is caused due to biallelic variants in *SLC2A2* (solute carrier family 2 member 2) or *GLUT2* (glucose transporter 2 protein) known to mediate bidirectional glucose transport [28]. Affected individuals with variants in this gene are also susceptible to noninsulin-dependent diabetes mellitus (NIDDM) [29]. The variant, c.589 G>C (in *SLC2A2*) observed in the present study (in P10) is a novel alternate variant for the first-ever reported variant in *SLC2A2* in the homozygous state which causes a different missense change at the same amino acid codon [28]. The reported variant, c.589G>A p.Val197Leu (in *SLC2A2*) has been in discussion since its first description as it was also reported in heterozygous state in African American women with diabetes mellitus (DM). Genotype–phenotype correlation by a recent study highlights that biallelic/nonfunctional variants show a complete spectrum of FBS including hepatonephromegaly owing to glycogen accumulation, renal tubular dysfunction, and hypophosphatemic rickets whereas subjects with single-heterozygote variants are susceptible to NIDDM owing to impaired sugar transport in the kidney [29]. Clinical features and biochemical findings (Supplementary Table S1) observed in the patient described in the present study (P10) are concordant with the complete phenotypic spectrum of FBS.

Diagnosis of rickets requires a multifaceted approach which begins with the collection of detailed family history, clinical evaluation, radiographic and biochemical investigations, and use of next-generation sequencing (NGS) to receive a definitive molecular diagnosis. Often, the clinical profile of monogenic rickets is similar to classical nutritional deficiency rickets, which leads to a delay in diagnosis and failure of treatment. However, in the present era, a low index of suspicion of genetic rickets and early deployment of genetic tests are necessary to achieve a rapid and accurate diagnosis. Recently, Marik et al. reported a cohort of 63 individuals affected with rickets and proposed a gene panel for the diagnosis of hypophosphatemic rickets [30]. Molecular diagnosis plays a major role in early intervention and helps in redirecting the treatment plan which would lead to significant improvement in clinical, biochemical, and radiological features.

## Conclusion

In this study, the authors have presented profiles of six different genetic forms of rickets including vitamin-D-dependent (VDDR1A, VDDR2A, and VDDR2B) rickets, hypophosphatemic rickets (XLDHR and HHRH), and a disease entity with rickets as one of the clinical features, Fanconi–Bickel syndrome (FBS). With the addition of six novel variants in four known genes (*CYP27B1*, *CYP2R1, SLC34A3,* and *SLC2A2*), the present study provides an update to the mutation spectrum.

### Supplementary Information

Below is the link to the electronic supplementary material.

Supplementary file1 (DOCX 272 KB)

Supplementary file2 (DOCX 24 KB)

Supplementary file3 (DOCX 13 KB)

Supplementary file4 (DOCX 16 KB)
