# Supplementary figures and images for: Exome Sequencing in Monogenic Forms of Rickets
Source: Indian J Pediatr. 2023 Jan 24;90(12):1182–90. doi: 10.1007/s12098-022-04393-9 (PMC10627992; doi:10.1007/s12098-022-04393-9)

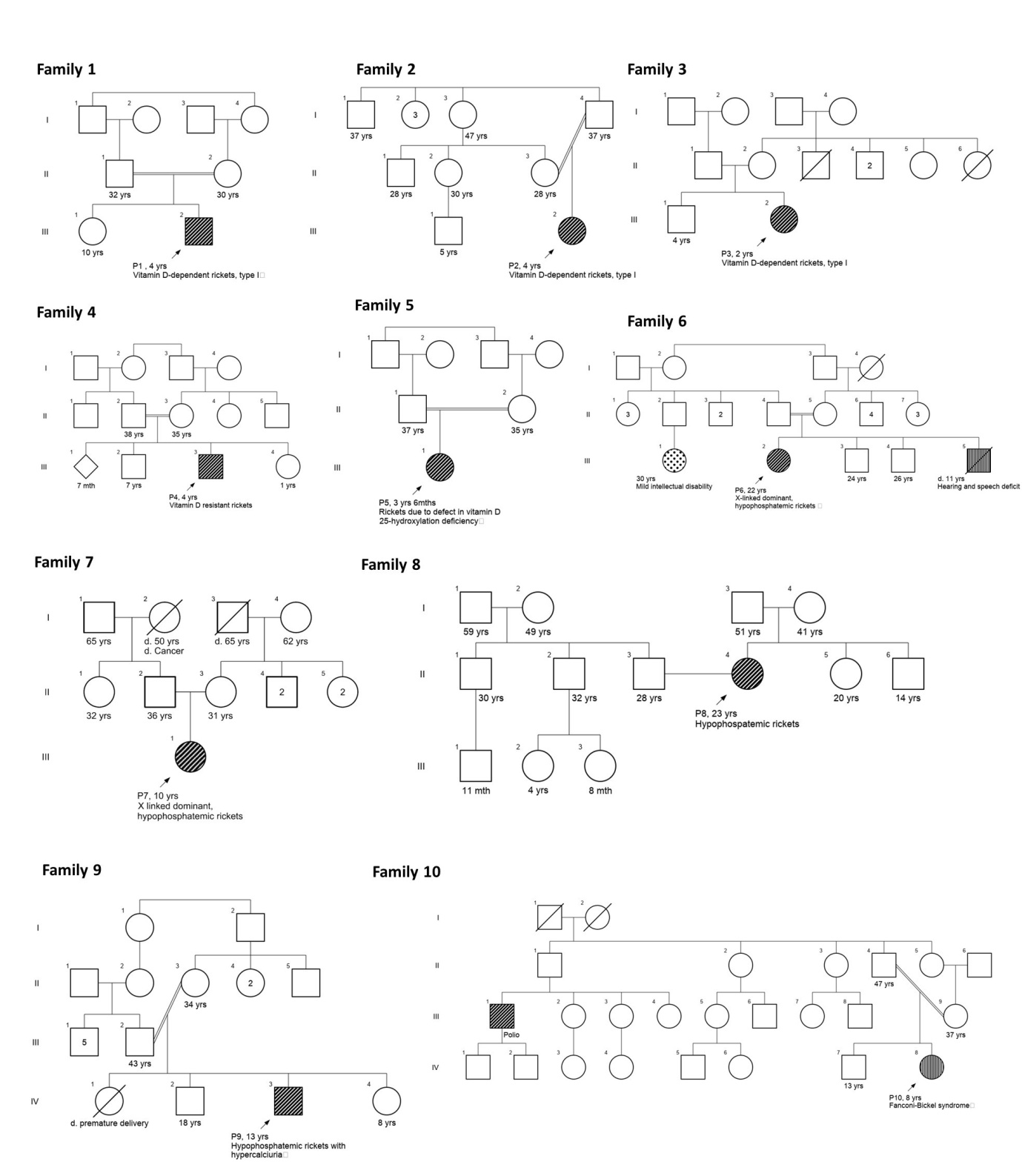


**Supplementary Fig. S1** Pedigrees of the families depicting affected subjects and phenotypes

Supplement: Supplementary file 1 — Supplementary file1 (DOCX 272 KB) [file 12098_2022_4393_MOESM1_ESM.docx]
