# Supplementary material for: Exome Sequencing in Monogenic Forms of Rickets
Source: Indian J Pediatr. 2023 Jan 24;90(12):1182–90. doi: 10.1007/s12098-022-04393-9 (PMC10627992; doi:10.1007/s12098-022-04393-9)
Supplement: Supplementary file 2 — Supplementary file2 (DOCX 24 KB) [file 12098_2022_4393_MOESM2_ESM.docx]

**Supplementary Material S1**

**Clinical details of individual families with a molecular diagnosis of rickets**

**Family 1**

A 4-y-old male child (P1), second born out of a second-degree consanguineous marriage, of Asian origin, presented with global developmental delay, short stature, and seizures with a history of recurrent respiratory infections which required hospitalization. There were no similar complaints in the family. He was born at term by elective cesarean section with a birth weight of 3.2 kg (-1.2 SDS) and cried immediately after birth. On the second day after birth, the child had involuntary movements suggestive of seizures which were treated promptly. The development was normal till 4 mo of age. At 4 mo, the patient had another episode of involuntary movements which resembled generalized tonic-clonic seizures. The frequency of these seizures was 3-4 times a day for which he was started on anti-epileptic drugs. Developmental milestones were delayed thereafter. Anthropometric examination at 4 y of age, suggested short stature with a height of 71 cm (-7 SDS). His weight and head circumference were 8.5 kg (-7 SDS) and 47 (-3 SDS), respectively. The child had dysmorphism (square-shaped face, maxillary prominence, coarse facies, protruded tongue, hypertelorism), rachitic rosary, anterior-lateral bowing of the legs, clubbing in the fingers, hepatosplenomegaly, and mild hypotonia. A skeletal survey revealed diaphyseal dysplasia and metaphyseal widening at the knee, protuberant costochondral junctions, bowing of humerus and tibia, oligodontia, delayed dentition, and no signs of fracture. His biochemical investigation (supplementary table S1) showed hypocalcemia, hypophosphatemia, normal vitamin D, elevated PTH, and ALP suggestive of calcipenic vitamin D-dependent rickets. He was treated with calcium supplements, calcitriol, and antiepileptic drugs (AED). Vitamin D was started at the age of 3 y 9 mo and continued till 6 y 4 mo. Eventually, the boy was seizure-free. Exome sequencing (ES) for the proband was performed which revealed a novel missense variant, c.974C>T p.(Thr325Met) in exon 6 in homozygous state in *CYP27B1* (NM_000785.4). This variant was not observed in gnomAD, ExAC, and the authors’ in-house database. In-silico tools (MutationTaster, SIFT and REVEL) predicted the variant to be damaging, thus confirming the diagnosis of vitamin-D-dependent rickets type IA (VDDR1A).

**Family 2**

A 4-y-old female child (P2) was conceived to a consanguineously married couple. The child was brought to clinical attention in view of limb deformity for the past 6 mo and swelling in the chest since 2 y of age. She was born at full term via caesarean section with a birth weight of 2.41 kg (-2.65 SDS). Her antenatal history was unremarkable. She cried immediately after birth and the feeding was normal. Postnatally, she had the first episode of seizures at 1 y of age, followed by the second episode after 1 wk and the third episode after 1 to 2 mo. Her investigations (Supplementary Table S1) were suggestive of hypocalcemia (other biochemical details unavailable). She was started on treatment with calcium supplements and antiepileptics. She had one more episode of seizures after 2 mo of post-treatment and no more episodes subsequently. On examination at 4 y of age, her height, weight, and head circumference were 87 cm (-2.5 SDS), 11 kg (-2.2 SDS), and 48.5 cm (-1.1 SDS), respectively. She was alert and active and had normal developmental milestones. Head-to-toe examination revealed dolichocephaly, low-set ears, pectus carinatum, bilateral widened wrist, and genu valgum. Her radiographs were suggestive of metaphyseal irregularity of the ulna and radius and enlargement of metaphysis at the knee along with severe osteopenia. Exome sequencing (ES) was opted for in view of hypocalcemic rickets and skeletal deformity which revealed a known biallelic truncating variant, c.1319_1325dup in *CYP27B1* (NM_000785.4) in the homozygous state was observed in the proband [1]. This variant was not observed in gnomAD, ExAC, and the authors’ in-house database. In-silico tools (MutationTaster, SIFT and M-CAP) predicted the variant to be disease-causing. Segregation analysis confirmed the heterozygous state of the variant in both parents thereby ascertaining the diagnosis of VDDR1A.

**Family 3**

A 2-y-old female child (P3) with normal birth history who was apparently well until 1 y of age, presented with a history of recurrent respiratory tract infection and loss of gross motor milestones since 1 y of age. She was brought to clinical attention when the mother noticed that the child had difficulty in getting up from supine position, inability to roll over, and preferred to lie only on the back. She could attain all the milestones at an age-appropriate timeline until 1 y 4 mo of age. A history of progressive weight loss in the child was also noted. She received the treatment of vitamin-D supplements until 1 y of age. At the age of 2 y, her height was 68 cm (-3.2 SDS), weight was 6.38 kg (-3 SDS) and head circumference was 44 cm (-2.6 SDS). Head-to-toe examination revealed open anterior fontanelle, frontal bossing, no dental abscess, presence of rachitic rosary, pectus carinatum, Harison sulcus, pot belly, widening of the wrist, and double malleoli. Systemic examination showed the presence of tachypnea and subcostal retractions in the respiratory system, hepatomegaly on abdomen palpation, bilateral lower limb hypotonia, and brisk deep tendon reflexes in neurological system assessment. Clinical radiographs were suggestive of osteopenia in long bones, widening of the growth plate, cupping of metacarpals, delayed bone age, fraying of metaphysis of long bones, and increased soft tissue density noted in the region of knee joint suggestive of uncalcified osteoid. Biochemical evaluation (supplementary table S1) showed normal vitamin D and calcium with low phosphorous and elevated ALP and PTH. Solo exome sequencing was offered to the family which resulted in the identification of one novel variant, c.1376G>A, and a known variant, c.1319_1325dup in compound heterozygous state in exon 8 of *CYP27B1* (NM_000785.4) [1]. The missense variant, c.1376G>A was observed in gnomAD in 4 individuals (allele frequency: 0.0000159) and the frameshift variant, c.1319_1325dup was observed in 56 individuals in the heterozygous state (allele frequency: 0.000223). These variants were not observed in the authors’ in-house database. In-silico tools (MutationTaster, SIFT and M-CAP) predicted the variant to be disease-causing. Segregation analysis confirmed the biallelic state of variants thereby confirming the diagnosis of VDDR1A.

**Family 4**

A 3 y 6 mo old female (P4) child born to a consanguineously married couple was presented with progressive bowing of legs since 1 y 6 mo of age. Her antenatal history was normal and she was born at full term via normal vaginal delivery. She had a birth weight of 3.6 kg (+0.66 SDS) and was admitted to NICU in view of suspected birth asphyxia. Her radiographs were suggestive of wide epiphyses and asymmetrical fraying (medial) of metaphyses of long bones. Her biochemical parameters were suggestive of low serum calcium, and phosphate levels with elevated ALP values (Supplementary Table S1). Analysis of ES data helped in the identification of two novel compound heterozygous variants, c.595C>T and c.1330G>C in exons 3 and 4 of *CYP2R1*(NM_024514.4). The first variant was observed in 3 individuals in the heterozygous state (allele frequency: 0.0000119) and the second variant, c.1330G>C was reported in 2 individuals in the heterozygous state (allele frequency: 0.00000799). These variants were not reported in the authors’ in-house database. In-silico tools (MutationTaster, SIFT and REVEL) predicted the variant to be disease-causing. The molecular diagnosis of vitamin-D-dependent rickets type 1B (VDDR1B) in the proband was confirmed after segregation analysis.

**Family 5**

A 4-y-old boy (P5), third born to a consanguineously married couple showed signs of wrist widening and leg deformity since age 1 y 6 mo. He was born at full term via caesarean section with a birth weight of 3.5 kg (-0.9 SDS). His antenatal history was uneventful with no perinatal complications; however, alopecia was noticed at birth. He had mild global developmental delay. At 4 y of age, his height was 76 cm (-6 SDS) and occipitofrontal circumference was 47 cm (-2.4 SDS). Head-to-toe clinical evaluation showed frontal bossing, complete alopecia, sparse eyebrows, carious teeth, pectus carinatum, and rachitic rosary, widening of wrist and ankles, joint laxity, and severe ‘double malleoli’ in ankles. Radiographs at age 4 y were suggestive of severe osteoporosis, lumbar kyphosis, delayed opacification of the epiphyses, frayed and irregular metaphyses of the knee, cupping of radius and ulna, and bending of the fibula. His laboratory findings (Supplementary Table S1) were suggestive of hypocalcemia, hypophosphatemia, and elevated ALP suggestive of rickets. He was started in treatment with calcium and vitamin D3 supplements; with no improvement in clinical and radiological features and hence ES was opted to investigate the genetic cause. Analysis of proband’s ES data lead to the identification of a known missense variant, c.1171C>T in homozygous state in exon 11 of *VDR* (NM_001017535.1) [2]. This variant was not observed in gnomAD and ExAC in any individuals. In-silico tools (MutationTaster, SIFT and M-CAP) predicted the variant to be damaging. His parents were carriers for this variant, and it was not observed in his unaffected brother thus confirming the diagnosis of vitamin D-resistant rickets, type IIA (VDDR2A).

**Family 6**

A 22-y-old girl (P7) born to a consanguineously married couple had genu varum since the age of 3 y. She was born at 9 mo gestation period via NVD with a birth weight of 3 kg (-1.2 SD). Her antenatal history was uneventful, and she cried immediately after birth. She had mild global developmental delay. On examination at 22 y of age, her height, weight, and occipitofrontal circumference were 145.7 cm (-2.6 SDS), 44 kg (-2.4 SDS), and 53.5 cm (-1 SDS), respectively. A clinical examination from head to toe showed telecanthus, bushy eyebrows, synorphis, low set ears, esotropia, and central corneal opacity with iridotomy in eyes, high arched palate, short trunk, bilateral pes planus, and everted left foot. Her systemic examination revealed unilateral vision loss, bilateral hearing loss, and primary amenorrhea. Her radiographs of lower limbs were suggestive of bowing of bilateral limbs, generalized osteoporosis, irregular metaphyses of the knee, and bending of the tibia and fibula. Biochemical test results were not available. ES of proband’s DNA sample revealed a known frameshift variant, c.1586_1586+1del in the heterozygous state of exon 14/intron 14 of *PHEX* (NM_000444.6) [3]*.* This variant was not observed in the DNA sample of the father, however, segregation analysis could not be completed due to the unavailability of the mother’s sample. This variant was not observed in gnomAD, ExAC, and the authors’ in-house database in any individuals. In-silico tools (Splice AI and Mutation taster) predicted these variants to be disease-causing, thus confirming the diagnosis of X-linked dominant hypophosphatemic rickets.

**Family 7**

A 10-y-old girl (P6) was presented with bilateral genu varum and difficulty in walking since 2 y and 6 mo of age. She was born to a non-consanguineous couple at full term via normal vaginal delivery (NVD) with a birth weight of 3.5 kg (+0.85 SDS). Her antenatal history was unremarkable at birth. Delay in the attainment of gross motor skills was observed as she could not sit and stand independently by the age of 1 y 2 mo and 2 y, respectively. She started walking at the age of 2 y and her cognition was normal. At 10 y of age, her height was 124.5 cm (-2.45 SDS), weight was 24 kg (-2 SDS) and head circumference was 52 cm (-1.33 SDS). On examination, she had mild wrist widening, bilateral genu varum, and intermalleolar and intercondylar distance was increased. Her radiographs showed bowing of the femora, generalized osteoporosis, frayed and irregular metaphyses of the knee, and fraying of the iliac wings. Her biochemical test (Supplementary Table S1) revealed hypophosphatemia and elevated ALP. She underwent bilateral epiphysiodesis surgical treatment for correction of bilateral genu varum. Solo ES performed in the proband helped in the identification of a known canonical splice variant, c.1482+5G>C in heterozygous state in intron 13 of *PHEX* (NM_000444.6) [4]. The variant was not observed in either of the parents, population databases (gnomAD and ExAC), and the authors’ in-house database. In-silico tools (Splice AI and MutationTaster) predicted these variants to be disease-causing. Thus the diagnosis of X-linked dominant, hypophosphatemic rickets (XLDHR) was confirmed.

**Family 8**

A non-consanguineously married couple sought pre-conceptional counseling in view of the clinical diagnosis of hypophosphatemic rickets in the female partner (P8). She was 23 y old and had genu valgum and abnormal gait since age three. On examination, her height was 127 cm (-5.2 SDS), weight was 39 kg (-2.9 SDS) and head circumference was 52.5 cm (-1.6 SDS). Her clinical features include mild contracture at both elbow joints, short stature, and bowing of both lower limbs. No other dysmorphism or abnormalities were seen. She had undergone multiple surgeries in the past for correction of genu varum. Radiographs at the age of 19 y were suggestive of osteopenia, coarse trabeculae, irregular metaphyses of the knee, and bending of the femur, tibia and fibula. Biochemical investigation (Supplementary Table S1) was suggestive of hypophosphatemia. ES analysis in her revealed a known stop gain variant, c.58C>T in *PHEX* (NM_000444.6) in the heterozygous state [5], this variant was not observed in any other individuals in the gnomAD population database and the authors’ in-house database. In-silico tools (MutationTaster, SIFT and REVEL) predicted the variant to be damaging. Thus the diagnosis of X-linked dominant, hypophosphatemic rickets was confirmed.

**Family 9**

A 13-y-old boy born (P9) to a consanguineously married couple was presented with short stature and an abnormal gait for 2 y. His antenatal history was uneventful and he was born at full term via normal vaginal delivery. His developmental milestones were age-appropriate. At the age of 13 y, his height, weight, and head circumference were 128 cm (-3.5 SDS), 30 kg (-4 SDS), and 50.5 (-3 SDS), respectively. Clinical examination revealed mild facial asymmetry, short trunk, bilateral hallux valgus, genu valgum, and sandal gap. His radiographs were suggestive of widened, distorted epiphyses, frayed and irregular metaphyses. His biochemical results (Supplementary Table S1) were suggestive of normal serum calcium and low phosphate levels with elevated ALP and normal PTH values. With the help of ES, a novel sequence variant, c.1336-11_1336-1del was observed in a homozygous state in intron 12 of *SLC34A3* (NC_000009.12). This variant was present in the heterozygous state in both parents. No individuals with this variant in the heterozygous state were found in gnomAD and ExAC. In-silico tools (Mutation taster, Splice AI)predicted this variant to be damaging, thus confirming the diagnosis of hypophosphatemic rickets with hypercalciuria (HHRH) in the proband.

**Family 10**

An 8-y-old girl (P10), born to a second-degree consanguineously married couple was referred to a genetics clinic in view of multiple fractures. She was unable to walk at the time of the evaluation. She was born at term with a birth weight of 2.5 kg (-2.25 SDS) and her antenatal history was uneventful. On clinical evaluation, she had trivial fractures at the femur and had callus formation at the site of the fracture. Her radiographs were suggestive of generalized osteopenia and extensive bowing of both upper extremities. In view of these features, a provisional diagnosis of osteogenesis imperfecta (OI) was ascertained. She did not have blue sclera and dentinogenesis imperfecta on assessment. On exome sequencing (ES), a novel homozygous missense variant, c.589G>C in exon 5 of *SLC2A2*(NM_000340.2) was observed which is known to cause Fanconi-Bickel syndrome (FBS). A re-examination of her radiographs showed cupping and fraying of metaphyses suggestive of rickets. Biochemical testing revealed glucosuria, hypophosphatemia, and elevated serum alkaline phosphatase (Supplementary Table S1) which are common features seen with Fanconi–Bickel syndrome. This variant was not observed in gnomAD and ExAC in any individuals. In-silico tools (MutationTaster, SIFT, and REVEL) predicted this variant to be disease-causing. Hence a diagnosis of FBS was confirmed in the proband.

**References**

1. Wang JT, Lin CJ, Burridge SM, et al. Genetics of vitamin D 1alpha-hydroxylase deficiency in 17 families. Am J Hum Genet. 1998;63:1694–702.

2. Whitfield GK, Selznick SH, Haussler CA, et al. Vitamin D receptors from patients with resistance to 1,25-dihydroxyvitamin D3: point mutations confer reduced transactivation in response to ligand and impaired interaction with the retinoid X receptor heterodimeric partner. Mol Endocrinol. 1996;10:1617–31.

3. Gaucher C, Walrant-Debray O, Nguyen T-M, Esterle L, Garabédian M, Jehan F. PHEX analysis in 118 pedigrees reveals new genetic clues in hypophosphatemic rickets. Hum Genet. 2009;125:401–11.

4. Tyynismaa H, Kaitila I, Näntö-Salonen K, Ala-Houhala M, Alitalo T. Identification of fifteen novel PHEX gene mutations in Finnish patients with hypophosphatemic rickets. Hum Mutat. 2000;15:383–4.

5. Francis F, Strom TM, Hennig S, et al. Genomic organization of the human PEX gene mutated in X-linked dominant hypophosphatemic rickets. Genome Res. 1997;7:573–85.
