# Supplementary material for: Exome Sequencing in Monogenic Forms of Rickets
Source: Indian J Pediatr. 2023 Jan 24;90(12):1182–90. doi: 10.1007/s12098-022-04393-9 (PMC10627992; doi:10.1007/s12098-022-04393-9)
Supplement: Supplementary file 3 — Supplementary file3 (DOCX 13 KB) [file 12098_2022_4393_MOESM3_ESM.docx]

**Supplementary Table S1** Laboratory parameters of all the affected individuals in the cohort

| Patient ID | Serum calcium (mg/dL) | Serum phosphate (mg/dL) | Serum alkaline phosphatase (IU/L) | Parathyroid hormone (pg/mL) | 25(OH)D (ng/mL) |
| --- | --- | --- | --- | --- | --- |
| P1 | 3.2 (low) | 3.3 (low) | 1081 (high) | 291 (high) | > 100 (normal) |
| P2 | 6 (low) | NA | NA | NA | NA |
| P3 | 8.9 (normal) | 2 (low) | 1882 (high) | 157.3 (high) | 51.03 (normal) |
| P4 | 8.2 (low) | 3 (low) | 403 (high) | NA | NA |
| P5 | 7.4 (low) | 2.4 (low) | 2056 (high) | NA | NA |
| P6 | NA | NA | NA | NA | NA |
| P7 | 9.4 (normal) | 2.2 (low) | 761 (high) | 53.8 (normal) | > 100 (normal) |
| P8 | 9 (normal) | 2.2 (low) | 193 (normal) | NA | > 100 (normal) |
| P9 | 9.6 (normal) | 3.4 (normal) | 399 (high) | 9.3 (low) | 31.24 (normal) |
| P10 | 8.9 (normal) | 1.4 (low) | 482 (high) | 22.3 (normal) | 32.17 (normal) |
| Reference ranges | 8.8–10.8 | 1–3 y: 3.8–6.5  4–11 y: 3.7–5.6  12–15 y: 2.9–5.4 > 15 y: 2.7–4.7 | < 300 | 15–65 | < 20: Deficient 20–30: Insufficient  > 30: Normal |
