# Supplementary material for: Exome Sequencing in Monogenic Forms of Rickets
Source: Indian J Pediatr. 2023 Jan 24;90(12):1182–90. doi: 10.1007/s12098-022-04393-9 (PMC10627992; doi:10.1007/s12098-022-04393-9)
Supplement: Supplementary file 4 — Supplementary file4 (DOCX 16 KB) [file 12098_2022_4393_MOESM4_ESM.docx]

**Supplementary Table S2** HGVS nomenclature of all the variants identified in the present study using Mutalyzer [1].

| Patient ID | HGVS nomenclature (with link to Mutalyzer) | Disease |
| --- | --- | --- |
|  |  |  |
| P1 | [NM_000785.4(CYP27B1_v001):c.974C>T; p.(Thr325Met)](about:blank) | Vitamin D dependent rickets  Type 1A (VDDR1A) |
| P2 | [NM_000785.4(CYP27B1_v001):c.1319_1325dup; p.(Phe443Profs*24)](about:blank) |  |
| P3 | [NM_000785.4(CYP27B1_v001):c.1376G>A ; p.(Arg459His)](about:blank) |  |
|  | [NM_000785.4(CYP27B1_v001):c.1319_1325dup; p.(Phe443Profs*24)](about:blank) |  |
| P4 | [NM_024514.4(CYP2R1_v001):c.595C>T ; p.(Arg199*)](about:blank) | Vitamin D dependent rickets  Type 1B (VDDR1B) |
|  | [NM_024514.4(CYP2R1_v001):c.1330G>C ; p.(Gly444Arg)](about:blank) |  |
| P5 | [NM_000376.3(VDR_v001):c.1171C>T ; p.(Arg391Cys)](about:blank) | Vitamin D dependent rickets  Type 2A (VDDR2A) |
| P6 | [NG_007563.2(PHEX_v001):c.1586_1586+1del](about:blank) | X-linked dominant hypophosphatemic rickets (XLDHR) |
| P7 | [NG_007563.2(PHEX_v001):c.1482+5G>C](about:blank) |  |
| P8 | [NM_000444.6(PHEX_v001):c.58C>T ; p.(Arg20*)](about:blank) |  |
| P9 | [NG_017008.2(SLC34A3_v001):c.1336-11_1336-1del](about:blank) | Hypophosphatemic rickets with hypercalciuria (HHRH) |
| P10 | [NM_000340.2(SLC2A2_v001):c.589G>C ; p.(Val197Leu)](about:blank) | Fanconi–Bickel syndrome (FBS) |

**Reference**

1. Lefter M, Vis JK, Vermaat M, den Dunnen JT, Taschner PEM, Laros JFJ. Next generation HGVS nomenclature checker. Bioinformatics. 2021;37:2811–7.
